# Expression of CD44 in Head and Neck Squamous Cell Carcinoma—An *In-Silico* Study

**DOI:** 10.1055/s-0043-1772459

**Published:** 2023-08-16

**Authors:** Loganathan Kavitha, Jayaseelan Vijayashree Priyadharsini, Deepthi Kattula, Umadevi Krishna Mohan Rao, Rajabather Balaji Srikanth, Manogaran Kuzhalmozhi, Kannan Ranganathan

**Affiliations:** 1 Department of Oral and Maxillofacial Pathology, Ragas Dental College and Hospital, ECR, Uthandi, Chennai, Tamil Nadu, India; Affiliated to The Tamil Nadu Dr. MGR Medical University, Guindy, Chennai, Tamil Nadu, India; 2 Clinical Genetics Lab, Centre for Cellular and Molecular Research (The Blue lab), Saveetha Dental College and Hospital, Saveetha Institute of Medical and Technical Sciences [SIMATS], Saveetha University, Chennai, Tamil Nadu, India; 3 Department of Conservative Dentistry and Endodontics, Ragas Dental College and Hospital, ECR, Uthandi, Chennai, Tamil Nadu, India; 4 Department of Oral and Maxillofacial Surgery, Balaji Dental Clinic, Tambaram West, Tambaram, Chennai, Tamil Nadu, India; 5 Department of Pathology, Aringnar Anna Memorial Cancer Research Institute, Kanchipuram, Karapettai, Tamil Nadu, India

**Keywords:** CD44, in-silico, cancer stem cell, HNSCC, UALCAN, TCGA

## Abstract

**Introduction** CD44, a multistructural and multifunctional transmembrane glycoprotein, is a promising cancer stem cell (CSC) marker that regulates the properties of CSCs, including self-renewal, tumor initiation, and metastasis, and confers resistance to chemotherapy and radiotherapy. The aim of the present study was to evaluate the gene and protein expression of CD44 and explore its prognostic value in head and neck squamous cell carcinoma (HNSCC).

**Methodology** The present observational study employs computational tools for analysis. The Cancer Genome Atlas Head-Neck Squamous Cell Carcinoma dataset (520 primary HNSCC and 44 normal tissues) from the University of Alabama at Birmingham Cancer platform was used to study the association of CD44 mRNA transcript levels with various clinicopathological characteristics of HNSCC including age, gender, tumor grade, tumor stage, human papillomavirus (HPV) status, p53 mutation status, and overall survival. The CD44 protein expression in HNSCC and normal tissues was ascertained using the National Cancer Institute's Clinical Proteomic Tumor Analysis Consortium Head-and-Neck cancer dataset (108 primary HNSCC and 71 normal tissues).

**Results** CD44 mRNA transcript and protein expression levels were significantly higher in HNSCC tissues than in normal tissues, and high CD44 expression was correlated with poor survival. CD44 was upregulated in Stage 1 and Grade 2 HNSCC compared with other stages and grades. Overexpression of CD44 was observed in HPV-negative and TP53-positive mutant status in HNSCC.

**Conclusion** The pleiotropic roles of CD44 in tumorigenesis urge the need to explore its differential expression in HNSCC. The study concludes that CD44 can be a potential diagnostic and prognostic biomarker for HNSCC and offer new molecular targets for CD44-targeted therapy for cancer management.

## Introduction

Head and neck squamous cell carcinoma (HNSCC) includes a heterogeneous group of malignancies that arise in the oral cavity, larynx, and pharynx. 1 2 HNSCC is the seventh most common cancer worldwide, accounting for over 800,000 new cases every year. 3 The high prevalence of HNSCC in regions such as Southeast Asia and Australia is associated with the use of specific carcinogen-containing products (tobacco smoking, areca nut chewing, and alcohol consumption), whereas increasing rates of oropharyngeal infection with HPV have contributed to the high prevalence of HNSCC in the United States and Western Europe. 1 Despite multimodal therapeutic approaches and advances in diagnostics, treatment, and surveillance, the 5-year progression-free survival (PFS) of HPV-negative patients with locoregionally advanced disease is approximately 40 to 50% and survival rates for recurrent/metastatic (R/M) disease have not significantly improved over the past 40 years. 4

The poor prognosis of HNSCC is primarily attributed to a lack of appropriate screening, late-stage diagnosis, and antineoplastic resistance of cancer stem cells (CSCs). CSCs constitute a minor fraction (1–3%) of the cells in primary tumors. The CSC hypothesis proposes that CSCs are subpopulations of tumor cells that are responsible for tumorigenesis, tumor differentiation, tumor maintenance, and invasion. The pool of CSCs with inherent characteristics of self-renewal and tumor heterogeneity seldom divides and remains undifferentiated. This facilitates the regeneration of new CSC promoting the development and perpetuation of several human malignancies. The characterization of CSC markers serves to identify novel therapeutic targets in HNSCC and can help in prognostication. 5

Several molecular protein biomarkers of HNSCC CSCs have been proposed, with CD44 being the most extensively validated for prognostic significance. 6 CD44, a cell surface receptor for hyaluronic acid and matrix metalloproteinases (MMPs) participates in intercellular interactions, cell adhesion, and cell migration. Upregulation of CD44 in HNSCC has been associated with increased loco-regional recurrence, metastasis, invasion, decreased overall survival, and increased resistance to radiotherapy. 7 We studied the significance of the CD44 gene and protein expression in HNSCC by using the Cancer Genome Atlas (TCGA) dataset accessed through the University of Alabama at Birmingham Cancer (UALCAN) portal.

## Data Source and Methods

The data on gene and protein expression of CD44 and its association with demographic features, clinicopathological characteristics, and survival outcomes were curated from UALCAN. UALCAN portal is an interactive and user-friendly web-based portal that enables researchers to access level 3 RNA-seq data from TCGA. It facilitates gene expression and survival analyses on around 20,500 protein-coding genes in 33 tumor types. It is built on PERL-CGI with high-quality graphics using javascript and Cascading Style Sheets. It is used to analyze relative gene transcript levels between tumors and normal samples, and the relationship between these levels and clinicopathological parameters. 8 Genomics data from the TCGA project include comprehensive molecular features of various cancer types. The large sample data of TCGA efficiently addresses the queries raised on tumor heterogeneity.

The mRNA transcript levels of CD44 in HNSCC and the corresponding normal controls in TCGA were reviewed using UALCAN. The clinicopathological parameters including age, gender, race, individual cancer stages, tumor grade, human papillomavirus (HPV), TP53 mutant status, and survival outcome of patients with primary HNSCC in TCGA-HNSCC were retrieved and grouped according to median CD44 mRNA expression. The CD44 protein expression analysis was done using data from Clinical Proteomic Tumor Analysis Consortium (CPTAC) and the International Cancer Proteogenome Consortium datasets available in UALCAN.

## Results

### Gene and Protein Expression of CD44 (The Cancer Genome Atlas and Clinical Proteomic Tumor Analysis Consortium Datasets in University of Alabama at Birmingham Cancer)

**Fig. 1:**
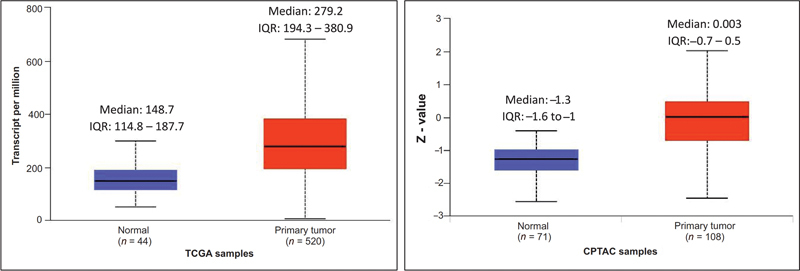
Gene and protein expression of CD44.

The TCGA dataset includes 520 cases of HNSCC and 44 healthy controls. Upregulation of median (IQR) *CD44* gene expression in HNSCC tumor samples (279.2) was significantly greater compared with normal subjects (148.7; *p* = 0.0001). The CPTAC dataset comprised 108 HNSCC samples and 71 healthy controls. Upregulation of CD44 protein expression in HNSCC tumor samples (median: 0.003) compared with normal subjects (median: –1.3); statistically significant ( *p* = 0.0008; Fig. 1 ).

### Association between CD44 Expression and Demographic Features (Age, Gender, and Race)

**Fig. 2:**
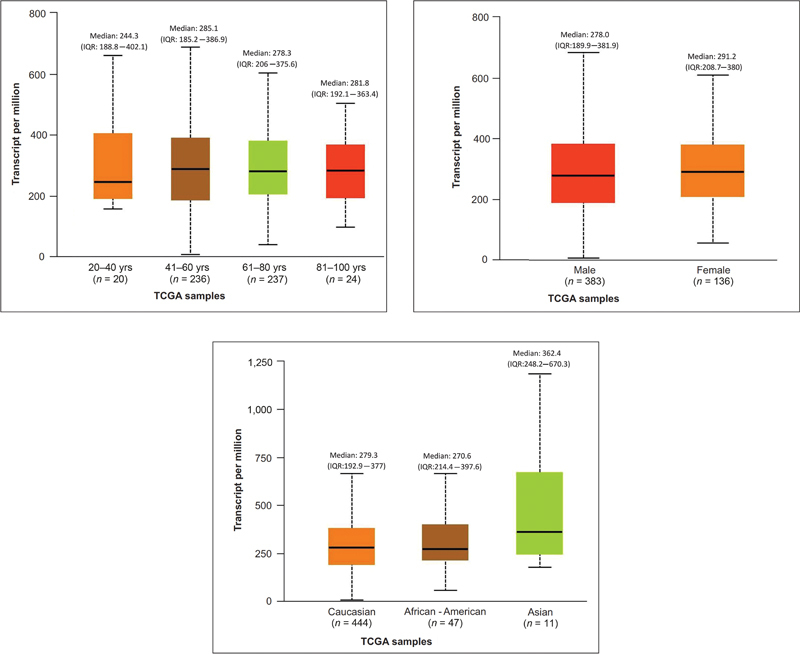
Association of CD44 and demographic features ( **[a]** age; **[b]** gender; **[c]** race).

Though HNSCC patients in the age group between 41 and 60 years ( *n* = 236) exhibited upregulation (median 285.1) of CD44 compared with the other age groups, it was not statistically significant. Similarly, females with HNSCC ( *N* = 136, 26%) showed overexpression of the *CD44* gene compared with males, but it was not statistically significant ( Fig. 2) .

The TCGA-HNSC dataset included 383 males (74%) and 136 females (26%). Among patients with HNSCC, females (median: 291.2) show overexpression of the *CD44* gene compared with males (median: 278), statistically not significant ( *p* = 0.2). Asians (median: 362.4), Caucasians (median: 279.2), and African-Americans (median: 270.6) show a significant overexpression of the *CD44* gene compared with the normal samples (median: 148.7), statistically significant ( *p* = 0.0006; Fig. 2 ).

### Effect of CD44 Expression on Clinicopathological Characteristics (Tumor Stage, Tumor Grade, Human Papillomavirus Status, and TP53 Mutation Status)

**Fig. 3:**
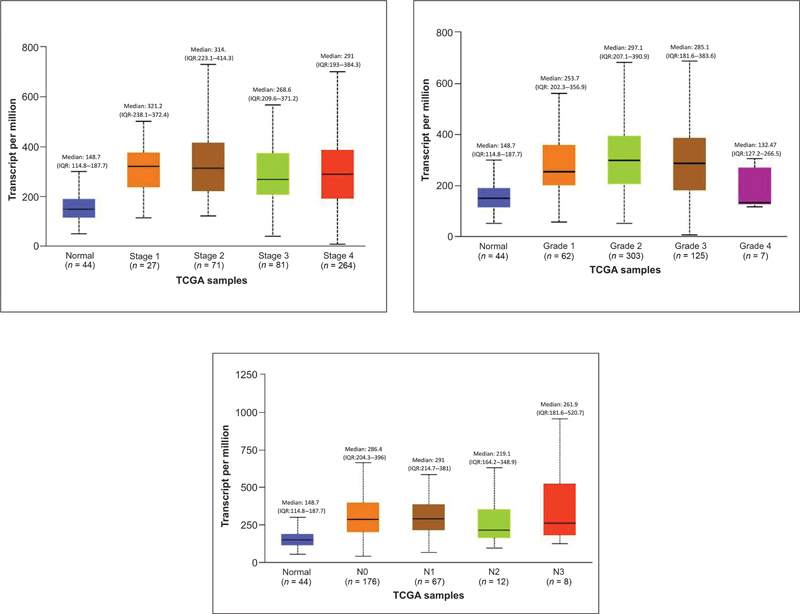
Effect of CD44 expression on tumor stage, tumor grade, and nodal status.

Overexpression of *CD44* gene in Stage 1 of HNSCC patients (Median: 321.2) compared with normal and other stages of HNSCC was statistically significant ( *p* = 0.0001). Grade 2 HNSCC tumors (median: 297.1) demonstrate an upregulation of CD44 compared with HNSCC patients with grade 1 (median: 253.7), and grade 3 (median: 285.1), and grade 4 (Median: 132.5) tumors and normal subjects (median: 148.7). HNSCC patients with N1 and N0 nodal status (median: 291; *p* = 0.0003 and 286.4; *p* = 0.0001, respectively) have significant CD44 upregulation compared with normal subjects (median: 147.8; Fig. 3 ).

**Fig. 4:**
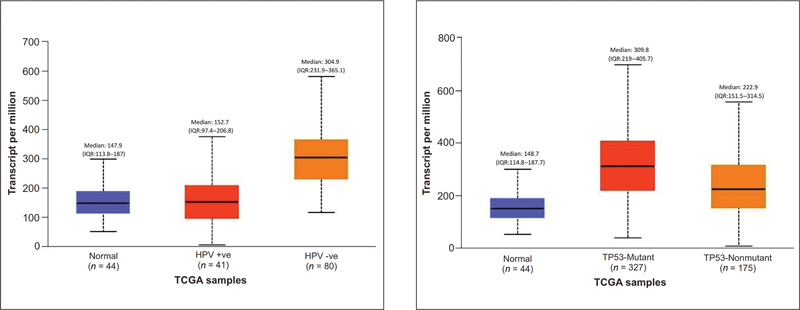
Effect of CD44 expression on HPV and TP53 status.

HNSCC tumors with negative HPV status (median: 304.9) exhibit greater *CD44* gene expression compared with patients with positive HPV status (median: 152.7) and normal subjects (median: 147.8), statistically significant ( *p* = 0.0001). TP53 mutant status positive HNSCC patients (median: 309.8) showed significant CD44 upregulation compared with TP53 nonmutant HNSCC patients (median: 222.9; Fig. 4 )..

### Association between CD44 Expression and Head and Neck Squamous Cell Carcinoma Patient Survival

**Fig. 5:**
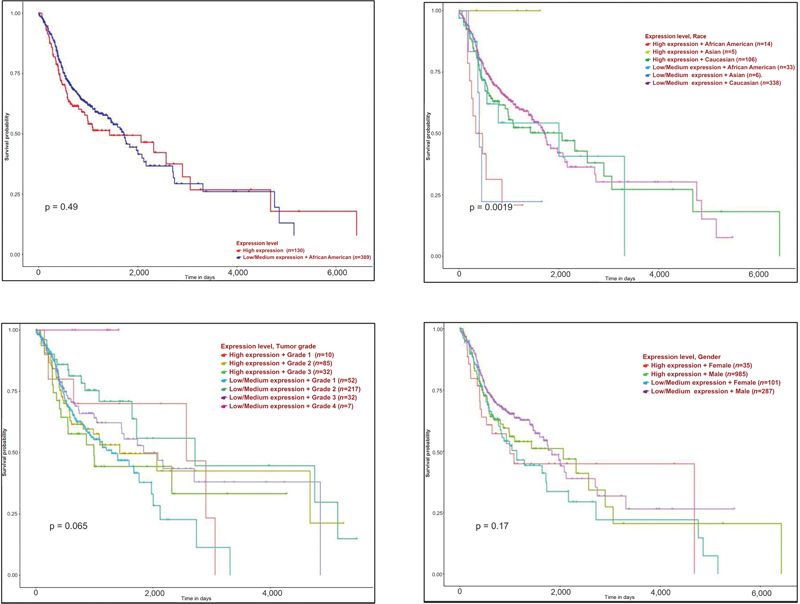
Association of CD44 expression and HNSCC patient survival.

High CD44 expression group had lower median survival compared with the low/medium expression group, but the difference was statistically not significant ( *p* = 0.49; Plot 1/Graph 1/ Fig. 1a–d ). Grade 3 tumors with high CD44 expression had a lesser median survival rate compared with Grade 1 tumors with low CD44 expression, statistically not significant ( *p* = 0.065). Irrespective of the differential expression of CD44, Caucasians had a higher median survival time compared with African Americans ( *p* = 0.0019) and had a higher median survival time compared with females ( *p* = 0.17; Fig. 5 ).

## Discussion

CD44, a cell membrane molecule, is a multistructural and multifunctional glycoprotein. It has a range of functions including cell adhesion (aggregation and migration), hyaluronan (HA) degradation, lymphocyte activation, lymph node homing, cell motility, cell migration, cell signaling (cell–cell and cell–matrix interactions), gene transcriptions, myelopoiesis, lymphopoiesis, angiogenesis, and release of cytokines because of its varied structure and distribution in the body tissues. The alternative splicing of variable exons of CD44 results in variants, which are denoted as CD44v, and the isoform with no variable exon in the mRNA as the standard isoform, CD44s. 9 In this study, we analyzed the expression patterns of the *CD44* gene and its association with demographic features, clinicopathological characteristics, and overall survival of patients with HNSCC by *in-silico* analysis.

CD44 is the major HA receptor, and CD44 bound to HA participates in tumor biological activities including tumor progression, metastasis, and proliferation. CD44 is the most frequently observed CSC marker in solid tumors. CD44 binding to HA regulated CSC survival, self-renewal, maintenance, and chemoresistance. 10

CD44 was first identified in lymphocytes. CD44s (standard form) is found in the lungs, epidermis, nervous system, stomach, pancreas, intestines, kidneys, urinary bladder, and cervix. CD44v (variants) are distributed in keratinocytes, lymphocytes, macrophages, and epithelial cells of the stomach, bladder, and cervix. 9 The data curated from the human protein atlas show the tissue-specific distribution of CD44 with high protein expression scores in the squamous epithelial cells of oral mucosa and tonsils and glandular cells of the salivary gland.

The pan-cancer profile of *CD44* gene expression in the Timer 2.0 platform shows significant upregulation of the *CD44* gene in cholangiocarcinoma, colon adenocarcinoma, esophageal carcinoma, glioblastoma multiforme, HNSCC, kidney chromophobe, kidney renal clear cell carcinoma, kidney renal papillary cell carcinoma, lung adenocarcinoma, pheochromocytoma and paraganglioma, prostate adenocarcinoma, rectum adenocarcinoma, stomach adenocarcinoma, thyroid carcinoma, and uterine corpus endometrial carcinoma. 11

Various studies support the significant overexpression of the *CD44* gene in human cancers. Wang et al reported that a high CD44 expression promotes lung cancer cell metastasis in vitro and in vivo through activation of ERK–ZEB1 signaling. 12 Gupta et al studied the immunohistochemical expression of CD44 in esophageal squamous cell carcinoma (ESCC) and its predisposing lesions (mild, moderate, and severe dysplasia and esophagitis). CD44 expression was significantly higher in ESCC as compared with dysplasia and esophagitis. 13 Using immunohistochemistry on tissue array, Zanjani et al evaluated CD44 expression in 206 renal tumor samples. Upregulation of CD44 expression was associated with aggressive behavior and poor prognosis in clear cell renal cell carcinoma (RCC) but not in papillary and chromophobe subtypes of RCC. 14 Wang et al in a meta-analysis of colorectal carcinoma (CRC) that included a total of 48 studies reported that the total CD44 isoform overexpression was significantly correlated with worse overall survival of patients with CRC. 15 CD44 modulates the aggressive phenotype of prostate cancer cells, by regulating the expression of PDK1 (pyruvate dehydrogenase) and PFKFB4 (6-phosphofructo-2-kinase/fructose-2,6-biphosphatase-4). 16 In the meta-analysis to ascertain the prognostic value of CD44 in RCC, high CD44 expression correlated with high Fuhrman grade, recurrence, microvascular invasion, and poor prognosis. 17 A study on the splice variants of CD44 demonstrated the distinct role of CD44v3 and CD44v6 in the progression of bladder cancer. 18 CD44v9 contributes to increased resistance to chemotherapy- or radiation-induced cell death in gastric carcinoma. CD44 also promotes epithelial-mesenchymal transition (EMT) in many cancer types such as colon cancer, gastric cancer, pancreatic cancer, prostate cancer, liver cancer, and glioma by upregulating mesenchymal markers and downregulating epithelial markers. 9

The data mined from the UALCAN platform show upregulation of *CD44* gene and protein expression in HNSCC compared with normal subjects. Sawant et al reported that serum CD44 concentration was found significantly high in patients with primary oral squamous cell carcinoma (OSCC) ( *n* = 64) as compared with healthy individuals ( *n* = 16; *p* < 0.001) and also in patients whose disease locally recurred ( *n* = 10) as compared with those did not recur ( *n* = 35; *p* = 0.0026). 19 Reategui et al characterized CD44v3 in HNSCC cell lines by reverse transcription polymerase chain reaction. The mean CD44v3 values for HNSCC tumors were elevated 4.5 times (0.43 ± 0.44) compared with the normal tissues (0.10 ± 0.11; *p* < 0.01). 20 In an immunohistochemical study conducted by Tamatani et al, CD44 and CD44v9 expressions were strongly detected in all OSCC tissues compared with normal epithelial cells. 21 Similarly in another study, CD44v6 expression was detected in the membrane of tumor cells in 94% of the OSCC cases ( *n* = 60). 22 In a comparative study conducted in Africa, CD44 expression was evaluated in the whole unstimulated saliva of patients with oral leukoplakia and OSCC by ELISA. A statistically significant overexpression of CD44 was demonstrated in OSCC patients compared with those with leukoplakia and healthy controls. 23 El-Gendi et al reported that papillary thyroid carcinoma (PTC; *n* = 30) showed higher CD44v6 expression than follicular thyroid carcinoma ( *n* = 10). 24 On the contrary, downregulation of the CD44 gene was associated with a poor prognosis of laryngeal carcinoma. 25

Age and gender did not show any significant association with CD44 expression. Similar results were reported in the studies conducted by Monteiro et al and Wagih et al. 22 23 Asian, Caucasian, and African-Americans showed significant overexpression of the CD44 gene expression compared with normal samples. Similar to our study results, Lee et al, observed that OSCC with positive mutant p53 expression displayed enhanced expression of CD44 ( *p* < 0.001). 26 A significant upregulation of the *CD44* gene in HPV-negative OSCC is observed in the present study. This is supported by Slavik et al who studied the prognostic significance of the mutual combination of CD44, EGFR, and p16 in HNSCC. The worst prognosis was seen in CD44 ^+^ /p16 ^−^ , EGFR ^+^ /p16 ^−^ , and EGFR ^+^ /CD44 ^+^ groups and in the EGFR ^+^ /CD44 ^+^ within the p16 negative cohort. 27

Upregulation of the *CD44* gene was seen in Stage I and Grade 2 irrespective of N1 or N0 nodal status. Poor survival was exhibited by high CD44 expression. Various study findings are congruent with the current study results. Mack and Gires observed that CD44s and CD44v6 were significantly upregulated in moderately differentiated SCC compared with poorly differentiated SCC and carcinoma *in-situ* . 28 In another study by Sawant et al, CD44v6 staining intensity was detected as significantly high in recurrent OSCC as compared with primary tumors ( *p* < 0.001), and it also correlated with poor survival ( *p* < 0.001). Furthermore, in combination, patients with increased CD44 concentration in serum and CD44v6 expression in tumors significantly correlated with local recurrence ( *p* < 0.001) and poor survival ( *p* < 0.001). 19 Okuyama et al and Fonseca et al showed that high CD44 expression was associated with cervical lymph node metastasis. 29 30 Tandon et al observed an upregulation of CD44 was detected in 48% of well-differentiated OSCC followed by a reduced expression in moderately differentiated and poorly differentiated OSCCs and the expression correlated with the tumor size (T) in 23% of cases and with lymph node metastases (N) in 42% of cases ( *p* ≤ 0.05). This suggests an association of CD44 with tumor aggressiveness and EMT related to loss of cell adhesion in a subset of OSCC. 31 Ortiz et al reported that CD44 immunoexpression was a significant predictor of lymph node metastasis. 32

In the present study, patients with Grade 3 HNSCC and high CD44 expression had a poor prognosis compared with patients with Grade 1 and Grade 2 tumors. de Moraes et al found that the 5-year cancer-specific survival rates for the CD44-negative and CD44-positive groups were 74 and 38%, respectively, although this difference did not reach statistical significance ( *p* = 0.052). 33 OSCC patients with CD44 immunoreactivity of >30% had 2.08-fold increased risk of death compared with those with <30% CD44 immunoreactivity. 34 Boxberg et al demonstrated that CD44 overexpression within the tumor core region and in lymph node metastases was identified as an independent prognostic factor for poor overall, disease-specific, and disease-free survival in subsets of patients with advanced OSCC. 35 Morand et al reported that CD44 expression was associated with increased DOI ( *p* = 0.018) and worse disease-specific survival ( *p* = 0.041). A 1 mm increase in DOI was correlated with 31.1% higher chance of metastasis. They explained that tumors with greater DOI showed stronger CD44 expression suggestive of EMT and CSC signaling enabling the tumor to invade and metastasize to the nearby lymph node. This suggests the importance of CD44 expression at the invasive tumor front in early OSCC and its relation with tumor invasion and regional metastasis. 36

Cohen et al, in a prospective study, showed that universal CD44 gross tissue staining and total protein (TP) levels ≥ 1 mg/mL demonstrated poorer PFS, with the latter also affecting OS. Poorer survival was associated with soluble CD44 ≥ 5.33 ng/mL and TP ≥ 1 mg/mL. Higher solCD44 and protein were significantly correlated with disease progression (solCD44: *p* = 0.008, protein: *p* = 0.003). Patients who died exhibited significantly higher solCD44 and protein as compared with those who lived (solCD44: *p* = 0.016, protein: *p* = 0.001). 37 In a systematic review and meta-analysis including 15 studies conducted by Chen et al, it was found that the total percentage of CD44 expression was 57.8%, with 49.3% in oral cancer patients, 66.4% in the pharynx, and 54.7% in larynx cancer patients expressing CD44. No significant correlation between clinical features and CD44 expression was revealed for oral cancer patients, but CD44 was shown to be associated with advanced T categories, worse N categories, higher tumor grades, and 5-year OS rates in patients with laryngeal and pharyngolaryngeal cancer. 10

The binding of CD44 to HA promotes the transcription of several MMPs and initiates focal matrix degradation. The intracellular domain of CD44 regulates a few target genes CD44, cyclin D1, MMP-9, HIF-2α, c-myc, and Twist1. Binding of cytoskeletal proteins ankyrin and ERM (ezrin, radixin, and moesin) to the cytoplasmic tail of CD44 links CD44 to actin-cytoskeleton with functions involved in the regulation of epithelial-mesenchymal transition in head and neck carcinogenesis. 38

Computational approaches have been beneficial for researchers as they provide access to an exhaustive collection of data and facilitate analysis within a short time and cost-effective way. As with any in-silico study, the current study also has limitations. There is a lack of enough clinical data to analyze the association between CD44 and HNSCC. The dataset selected had individuals from different ethnic groups which made data analysis and interpretation to be more generic rather than specific. CD44 can undergo isoform switching in tumor cells. The association of CD44 variants with the clinicopathological characteristics of the study population is not addressed. However, there are several in-silico studies done to identify novel genetic markers which can be applied for population-based screening. 12 39 40 41 42

## Summary and Conclusion

The present study integrated the data obtained from public databases to gain a deeper insight into the role of CD44 in HNSCC. The gene and protein expression of CD44 was up-regulated in HNSCC and significantly correlated with the clinicopathologic characteristics. Upregulation of CD44 was associated with poor prognosis of HNSCC patients suggesting its role as a valuable diagnostic and prognostic marker in HNSCC. The pleiotropic roles of CD44 in carcinoma offer new molecular targets for CD44-targeted therapy for cancer management. However, further studies should be conducted in-vivo and in-vitro to investigate the functions and mechanisms of CD44 in HNSCC.
